# Bioinformatics-Guided Analysis Uncovers AOX1 as an Osteogenic Differentiation-Relevant Gene of Human Mesenchymal Stem Cells

**DOI:** 10.3389/fmolb.2022.800288

**Published:** 2022-02-23

**Authors:** Lingtong Sun, Jianfei Ma, Juan Chen, Zhijun Pan, Lijun Li

**Affiliations:** ^1^ Affiliated Hangzhou Xixi Hospital, Zhejiang University School of Medicine, Hangzhou, China; ^2^ Key Laboratory of Image Information Processing and Intelligent Control, School of Artificial Intelligence and Automation, Huazhong University of Science and Technology, Wuhan, China; ^3^ Department of Orthopedics Surgery, The Second Affiliated Hospital, Zhejiang University School of Medicine, Hangzhou, China; ^4^ Orthopedics Research Institute of Zhejiang University, Hangzhou, China; ^5^ Key Laboratory of Motor System Disease Research and Precision Therapy of Zhejiang Province, Hangzhou, China

**Keywords:** bioinformatics, human bone marrow mesenchymal stem cells, osteogenic differentiation, novel, prediction

## Abstract

**Background:** The available therapeutic options of bone defects, fracture nonunion, and osteoporosis remain limited, which are closely related to the osteogenic differentiation of bone marrow–derived mesenchymal stem cells (BMSCs). Thus, there remains an urgent demand to develop a prediction method to infer osteogenic differentiation–related genes in BMSCs.

**Method:** We performed differential expression analysis between hBMSCs and osteogenically induced samples. Association analysis, co-expression analysis, and PPI analysis are then carried out to identify potential osteogenesis-related regulators. GO enrichment analysis and GSEA are performed to identify significantly enriched pathways associated with AOX1. qRT-PCR and Western blotting were employed to investigate the expression of genes on osteogenic differentiation, and plasmid transfection was used to overexpress the gene AOX1 in hBMSCs.

**Result:** We identified 25 upregulated genes and 17 downregulated genes. Association analysis and PPI network analysis among these differentially expressed genes show that AOX1 is a potential regulator of osteogenic differentiation. GO enrichment analysis and GSEA show that AOX1 is significantly associated with osteoblast-related pathways. The experiments revealed that AOX1 level was higher and increased gradually in differentiated BMSCs compared with undifferentiated BMSCs, and AOX1 overexpression significantly increased the expression of osteo-specific genes, thereby clearly indicating that AOX1 plays an important role in osteogenic differentiation. Moreover, our method has ability in discriminating genes with osteogenic differentiation properties and can facilitate the process of discovery of new osteogenic differentiation–related genes.

**Conclusion:** These findings collectively demonstrate that AOX1 is an osteogenic differentiation-relevant gene and provide a novel method established with a good performance for osteogenic differentiation-relevant genes prediction.

## Introduction

The rate of bone defects, fracture nonunion, and osteoporosis incidence continues to rise, and available therapeutic options remain limited. These diseases, closely related to the osteogenic differentiation of bone marrow–derived mesenchymal stem cells (BMSCs), have been confirmed by numerous studies (Marongiu et al., 2020; Xiao et al., 2020; Xiong et al., 2020; Jiang et al., 2021).

BMSCs possess self-renewal capabilities and the potential to differentiate into a variety of cell types, including osteoblasts, chondrocytes, and adipocytes (Bianco et al., 2001; Liao et al., 2017). As a key contributor to the bone formation, BMSCs are regulated by genetic factors (Zhang et al., 2018). However, relying solely on the experimental identification of genes that regulate osteogenic differentiation of BMSCs is generally costly and time-consuming, which leads to an urgent demand to develop a prediction method to infer osteogenic differentiation–related genes in a short time.

High-throughput sequencing including RNA-seq and microarray is a novel technique, which plays an important role in the exploration for genome‐level differences and is providing valuable insights into the landscape of the identification of key genes and functional pathways associated with osteogenic differentiation in BMSCs (Fan et al., 2020). In our study, we performed a bioinformatics-guided methodology to identify potential regulators of osteogenic differentiation. We first identified 25 upregulated and 17 downregulated genes between hBMSCs and osteogenically induced samples. Association analysis, co-expression analysis, and PPI analysis are then carried out to infer potential regulators. As a result, AOX1 is identified as a new potential regulator. Association analysis, GO enrichment analysis, and GSEA show that AOX1 is significantly associated with osteogenic differentiation.

Aldehyde oxidase (AOX) is a complex enzyme present in the cytosol of eukaryotes (Garattini et al., 2008; Garattini et al., 2009). In humans, only one *AOX1* functional gene is present (Terao et al., 2006), which is expressed predominantly in the liver but detectable amounts are also found in other organs (Garattini and Terao, 2012). Although its physiological substrates are still unclear, AOX1 is suggested to have an important role in the metabolism of drugs and xenobiotics in the liver (Pryde et al., 2010; Garattini and Terao, 2011). However, its role with respect to the regulation of osteogenic differentiation has not previously been reported.

In this study, we examined the role of AOX1 in the osteogenic differentiation of human BMSCs (hBMSCs) through bioinformatics analysis and experimental verification. The results indicate that AOX1 plays an important role in the osteogenic differentiation *via* multiple pathways, meanwhile providing a novel method for the further study of osteogenic differentiation of human bone marrow–derived mesenchymal stem cells.

## Result

### Identification of Differentially Expressed Genes That Are Associated With Osteogenic Differentiation

**FIGURE 1:**
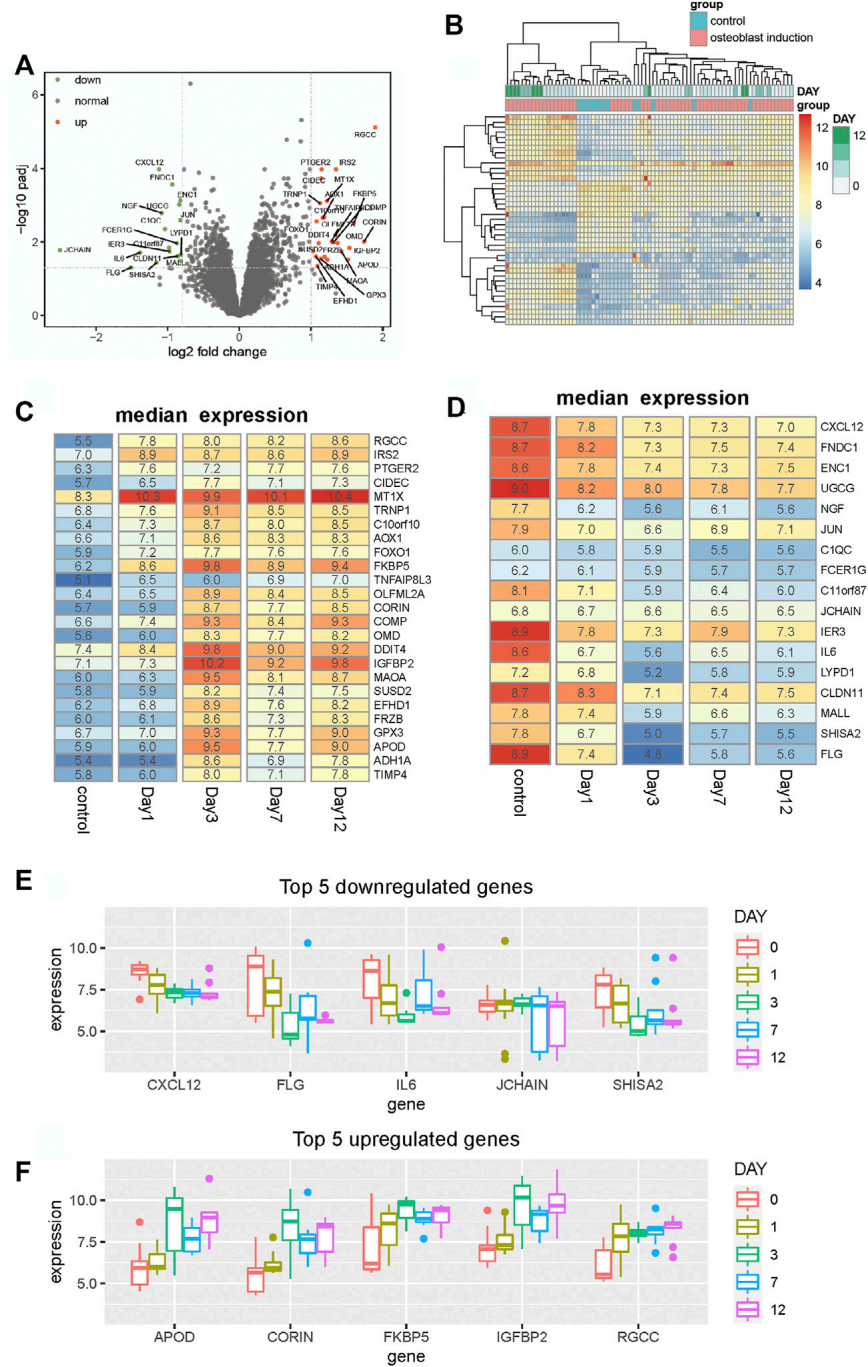
Identification of differentially expressed genes between hBMSCs and osteogenically induced samples. **(A)** Volcano plot shows the differentially expressed genes based on fold change and BH-adjusted *p-*value. Upregulated and downregulated genes are displayed using red and blue nodes, respectively. **(B)** Heat map shows the expression pattern of these differentially expressed genes. Samples are annotated into different groups. Day means the time of osteogenically induced samples in the differentiation process. **(C,D)** Heat map shows the median expression of upregulated and downregulated genes respectively for samples in different differentiation stages. **(E,F)** Boxplot shows the top five downregulated and upregulated genes respectively in the osteogenic process.

To characterize the process of osteogenic differentiation, we performed differential expression analysis between hBMSCs and osteogenically induced samples using the limma R package (Ritchie et al., 2015). As a result, 42 genes are identified to be differentially expressed. Among all of these differentially expressed genes, 25 genes are upregulated and the remaining 17 genes are downregulated in the osteogenically induced samples (Figure 1A). Clustering analysis of samples based on these differentially expressed genes shows that samples within the same group tend to be clustered together (Figure 1B), suggesting that these differentially expressed genes can differentiate between the control group and osteogenically induced samples. Furthermore, in the osteogenically induced group, samples in the advanced stages of the osteogenic process are found to be clustered with each other, suggesting that the osteogenic stage can indeed affect the expression of these differentially expressed genes.

To analyze the expression variation of these differentially expressed genes during the osteogenic process and identify to which extent the osteogenic stage can affect the expression of these genes, we analyzed the expression of these differentially expressed genes of different osteogenic stages. The median expression of upregulated and downregulated genes is calculated respectively for samples within the same osteogenic stage. Among all upregulated genes, we found that nearly half of these genes tend to display expression variation after day 1, such as OLFML2A, CORIN, MAOA, SUSD2, FRZB, APOD, and ADH1A (Figure 1C). Moreover, in the downregulated genes, we observed similar variations. Some genes start downregulating after the osteogenic stage day 1 and then maintain stability in the following stages (Figure 1D). Among these differentially expressed genes, CXCL12, FLG, IL6, JCHAIN, and SHISA2 are the top five downregulated genes, and APOD, CORIN, FKBP5, IGFBP2, and RGCC are the top five upregulated genes. The expression profiles of these genes, presented in Figures 1E,F, show that the expression is distinct between different stages. These results collectively suggest that the osteogenic stage can indeed affect the expression of these genes, especially in the early stage of the osteogenic process.

### Analysis of Osteoblast-Related Pathway in the Differentiation Process

**FIGURE 2:**
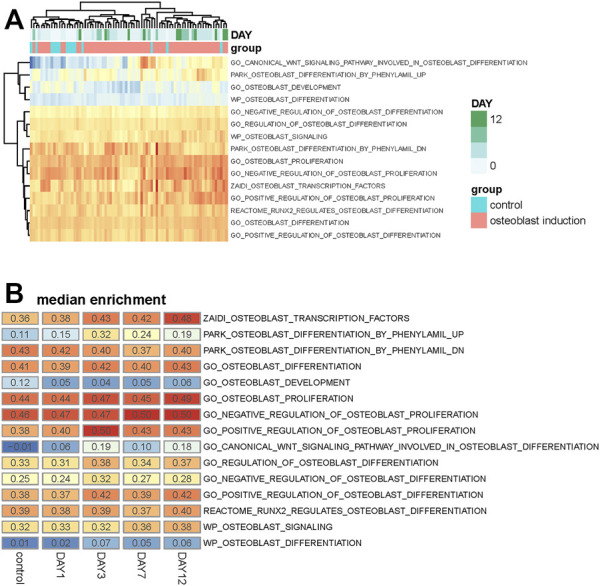
Analysis of osteoblast-related pathways in the osteogenic process. **(A)** Heat map shows the enrichment score of 15 osteoblast-related pathways across all samples. Samples are annotated into different groups and days. **(B)** Heat map shows the median value of the enrichment score of these osteoblast-related pathways across different osteogenic stages.

Differentially expressed genes with osteogenic differentiation are analyzed, but the activity of osteoblast-related pathways during the osteogenic process is unknown. To analyze whether these osteoblast-related pathways are activated or inhibited, we collected 15 osteoblast-related pathways from the Molecular Signatures Database (MSigDb) (Subramanian et al., 2005). Single-sample gene set enrichment analysis is used to quantify the activity of osteoblast-related pathways and calculate an enrichment score for each pathway of individual sample. Similar with differentially expressed genes, we found that samples within the same group and stage tend to be clustered together based on the enrichment score of these osteoblast-related pathways (Figure 2A), suggesting that osteogenic stages can indeed affect the activity of these pathways.

**FIGURE 3:**
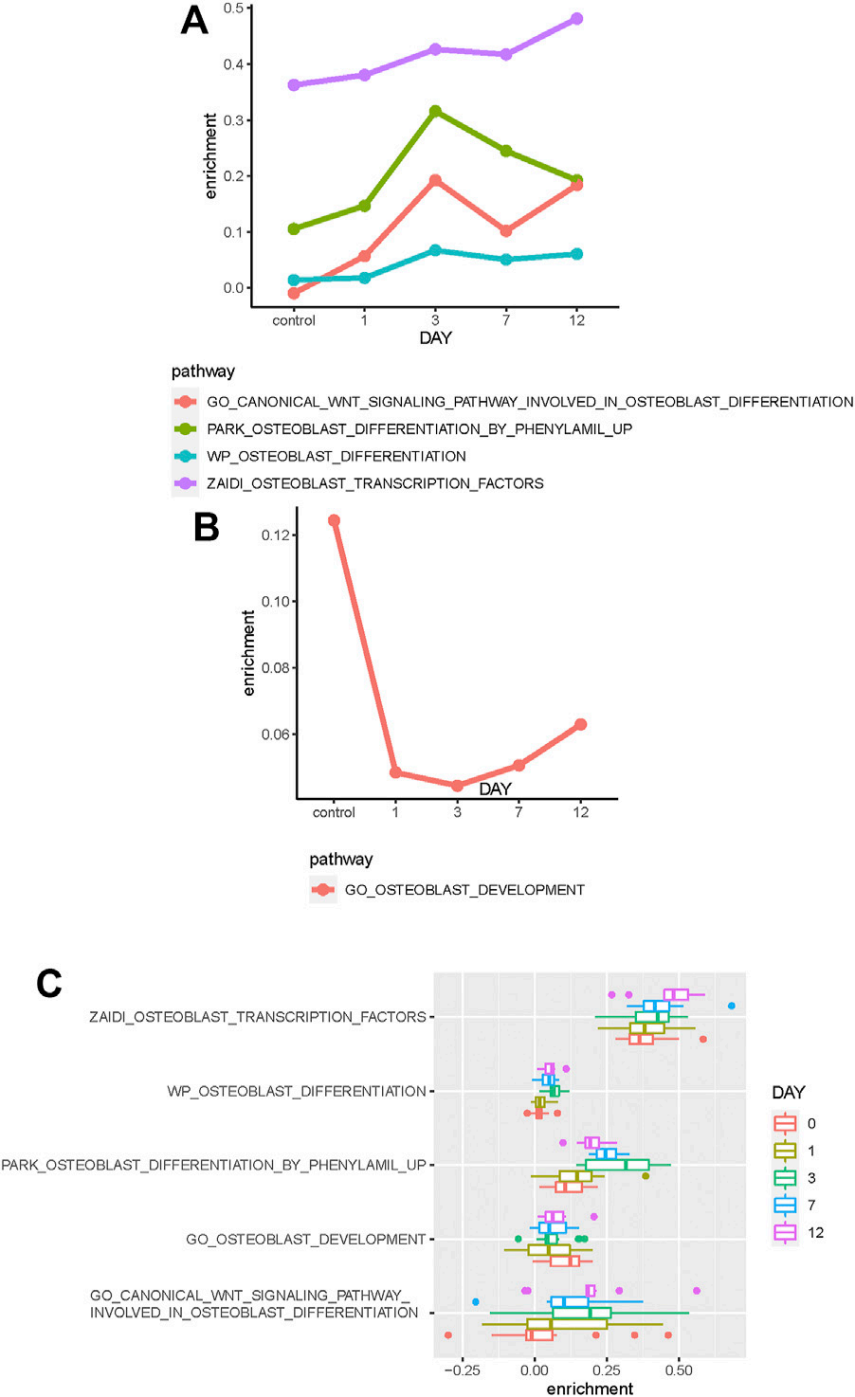
Identification of differentially enriched pathways. **(A)** Four osteoblast-related pathways tend to be upregulated in the osteogenic process. **(B)** One osteoblast-related pathway tends to be downregulated in the osteogenic process. **(C)** Boxplot shows these differentially enriched pathways of samples. Samples are divided into different groups based on osteogenic days.

The median enrichment score of these osteoblast-related pathways for different osteogenic stages is calculated to identify differentially enriched pathways. We found that most of these osteoblast-related pathways tend to display upregulated trends during the osteogenic process (Figure 2B). Four representative pathways are then identified to be upregulated, in which three are associated with osteogenic differentiation and one is associated with osteoblast transcription factors (Figure 3A), suggesting that osteoblast differentiation–related pathways are specifically enhanced and activated during the osteogenic process. Interestingly, one pathway associated with osteoblast development is found to be downregulated during the osteogenic process (Figure 3B), suggesting that the differentiation and development of hBMSCs are mutually exclusive during the osteogenic process. The enrichment pattern of these pathways is presented in Figure 3C.

### Selection of Candidate Genes That Are Associated With Osteogenic Differentiation

**FIGURE 4:**
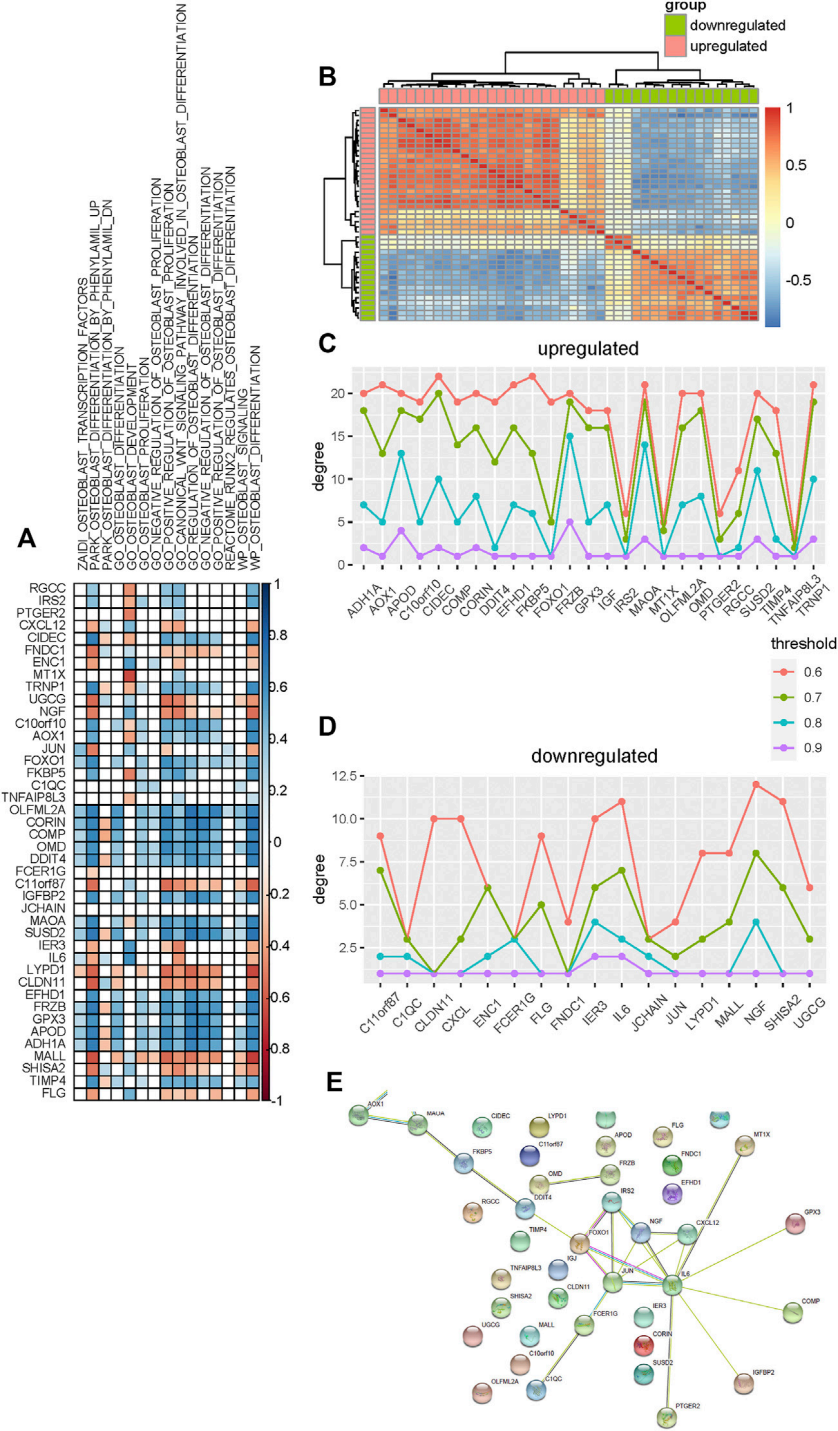
Selection of candidate genes that are significantly associated with the osteogenic process. **(A)** Association analysis between these differentially expressed genes and osteoblast-related pathways. Blue and red colors indicate the positive and negative correlations, respectively. Blank indicates that there is no significance. **(B)** Heat map shows the co-expression pattern of these differentially expressed genes. Association is evaluated using the Pearson correlation coefficient. **(C,D)** Degree of upregulated and downregulated genes in the co-expression network constructed using different thresholds. **(E)** Protein–protein interaction network of these differentially expressed genes is constructed.

To identify candidate genes that are significantly associated with the osteogenic process, we performed association analysis between these differentially expressed genes and osteoblast-related pathways. Most of these genes are found to be positively or negatively associated with osteoblast-related pathways (Figure 4A). Furthermore, analysis shows that upregulated genes tend to be positively associated with these pathways, such as C10orf10, AOX1, OLFML2A, CORIN, COMP, OMD, DDIT4, MAOA, and SUSD2, suggesting that these genes are significant determinants in promoting the osteogenic process. Analysis of downregulated genes shows that they are more likely to be negatively correlated with these osteoblast-related pathways, such as UGCG, NGF, C11orf87, LYPD1, CLDN11, and MALL, suggesting significant regulation of these genes in inhibiting the osteogenic process.

Co-expression analysis is then used to identify candidate genes associated with osteogenic differentiation. We evaluated the expression association among these differentially expressed genes based on the Pearson correlation coefficient and constructed the co-expression network for upregulated and downregulated genes respectively (Figure 4B). To make sure that the co-expression network is reliable and hub genes identified indeed capture the topological structure of the network, the network is constructed based on a varied association threshold from 0.6 to 0.9. Degree analysis for these upregulated co-expression networks shows that MAOA, FRZB, CIDEC, TRNP1, and APOD are hub genes (Figure 4C). Analysis for downregulated co-expression networks shows that NGF, IL6, and IER3 are hub genes (Figure 4D).

Additionally, the protein–protein interaction network is also used to identify hub genes that may be associated with the osteogenic process. We constructed a protein–protein interaction network for these differentially expressed genes using the STRING database (Szklarczyk et al., 2019) and then rank these genes by degree. As a result, nine genes are identified as hub genes based on the degree (Figure 4E). Among these hub genes, ADH1A, AOX1, MAOA, IRS2, and FOXO1 are upregulated and JUN, NGF, IL6, and CXCL12 are downregulated.

Based on these results, a few genes are identified as potential targets that may promote or inhibit osteogenic differentiation during the osteogenic process. Among these candidate genes, some of them are involved in osteoblast-related pathways, and some genes have been previously studied to be associated with osteogenic differentiation. These genes are then removed from the candidate gene set. In the remaining candidate genes, AOX1 has not yet been studied extensively and no literature reports its relationship with osteogenic differentiation. Due to the strong statistical evidence of AOX1 in the osteogenic process, it is necessary and important to elucidate the interaction between AOX1 and osteogenic differentiation.

### AOX1 Plays an Important Role in the Process of Osteogenic Differentiation

**FIGURE 5:**
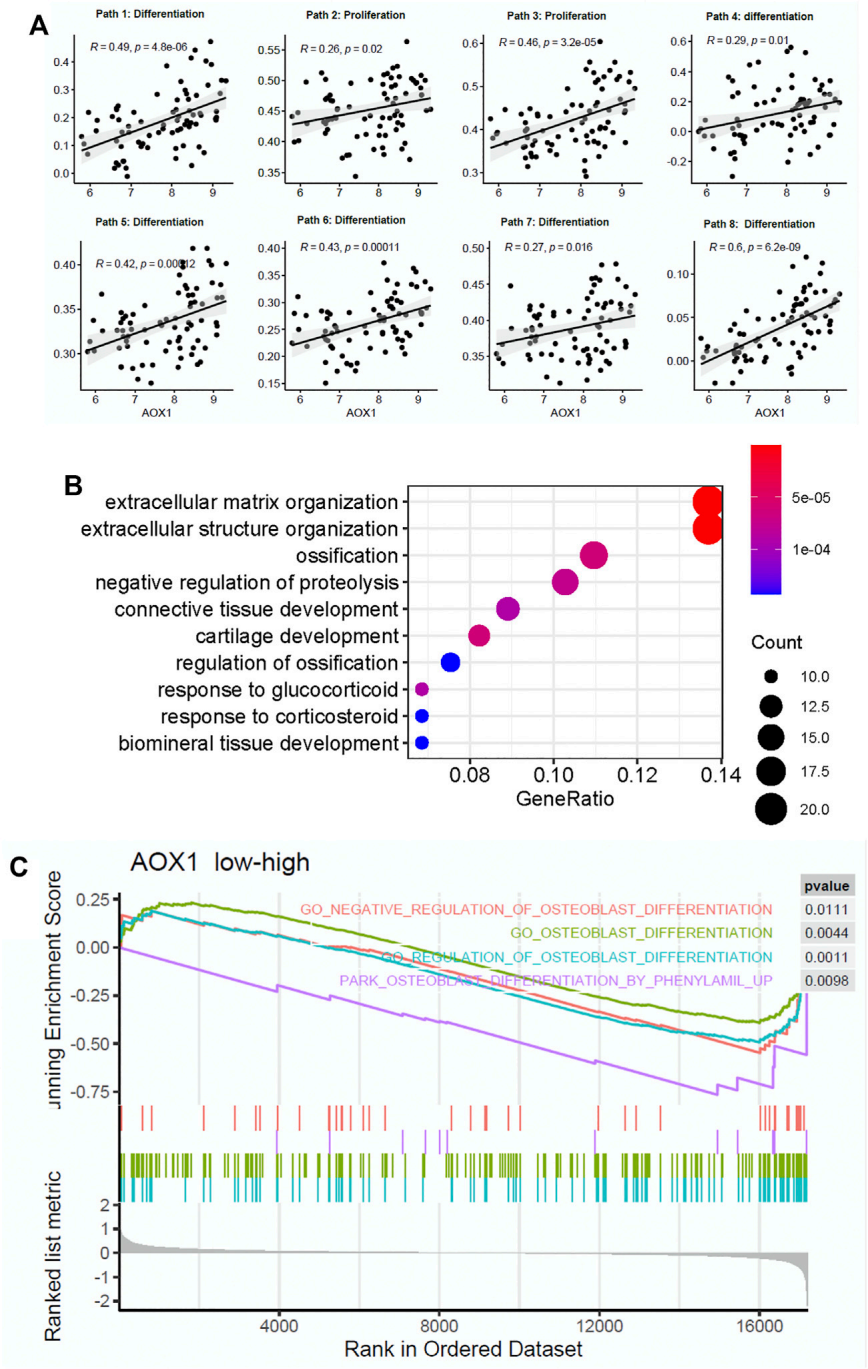
AOX1 is a potential gene positively regulating the osteogenic process. **(A)** Point diagram shows the positive correlation between AOX1 expression and the enrichment score of osteogenic pathways (the detailed information of osteogenic pathways are presented in Supplementary Table). **(B)** Gene ontology enrichment analysis of these differentially expressed genes between the high AOX1 expression group and the low expression group. **(C)** Gene set enrichment analysis between the high AOX1 expression group and the low expression group.

To determine the potential role of AOX1 in regulating the osteogenic process, we analyzed the association between AOX1 and osteoblast-related pathways. We found that AOX1 expression is positively associated with the enrichment of eight osteoblast-related pathways, in which two are associated with osteoblast proliferation and six are associated with osteogenic differentiation (Figure 5A), suggesting that AOX1 is a potential regulator in the osteogenic process.

Additionally, we divided samples into high AOX1 expression and low expression groups based on the median of AOX1 expression. Differential expression analysis is applied between these two groups to identify differentially expressed genes associated with AOX1. As a result, 152 genes are identified as differentially expressed between these two groups. The GO enrichment analysis is then used to identify whether these differentially expressed genes are enriched in some specific pathways. The GO enrichment analysis shows that these genes are enriched in ossification and regulation of ossification, further demonstrating the regulation of AOX1 in the osteogenic process. The top 10 enriched pathways are presented in Figure 5B.

Apart from the GO enrichment analysis, the gene set enrichment analysis is used to determine whether some pathways are enriched in a specific AOX1 group. All genes ranked by fold change between AOX1 groups are inputted into the gene set enrichment analysis, and 15 osteoblast differentiation–related pathways are inputted to analyze whether these genes are enriched in specific pathways. As a result, four osteoblast differentiation–related pathways are found to be enriched in the high-AOX1 expression group (Figure 5C). In conclusion, all these results suggest that AOX1 is indeed a potential regulator in the process of osteogenic differentiation.

### AOX1 Level Was Higher and Increased Gradually in Differentiated BMSCs Compared With Undifferentiated BMSCs

**FIGURE 6:**
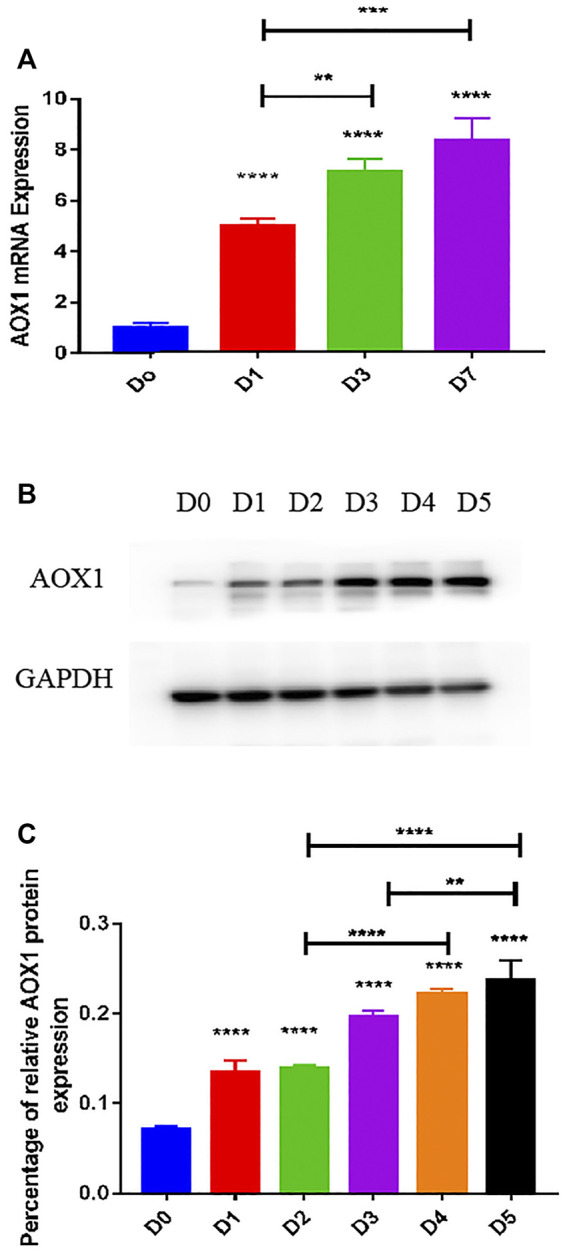
Endogenous AOX1 mRNA expression and the endogenous AOX1 protein expression on osteogenic differentiation of hBMSCs. **(A)** Endogenous expression of AOX1 mRNA was determined by qPCR at days 0, 1, 3, and 7 of osteogenic differentiation. **(B)** Endogenous expression of AOX1 protein was determined by Western blotting analysis at days 0, 1, 2, 3, 4, and 5 of osteogenic differentiation **(C)** Relative quantitative analysis of Western blot analyses for AOX1, data are used by percentage and expressed as the mean ± SD.

To determine the expression level of AOX1 associated with osteogenic differentiation of MSCs, we examined endogenous AOX1 expression in hBMSCs at days 0, 1, 3, and 7. Compared with undifferentiated BMSCs, both the mRNA (Figure 6A) and protein expression (Figures 6B,C) of AOX1 were higher and increased gradually in osteogenic differentiation**.**

### AOX1 Overexpression in hBMSCs

**FIGURE 7:**
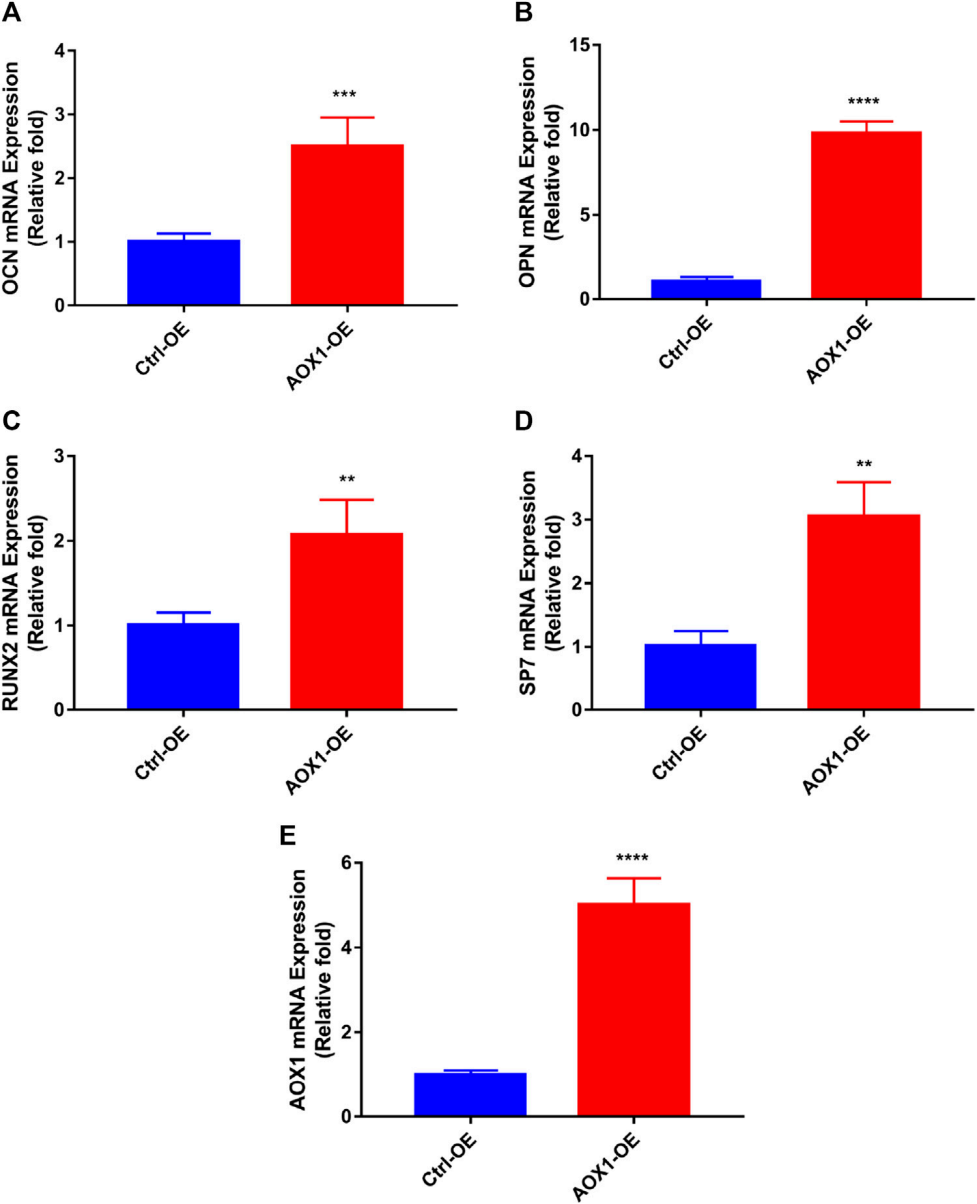
Effects of AOX1 overexpression on osteo-specific genes. **(A–D)** Relative mRNA expression of osteo-specific genes and **(E)** AOX1 on day three of osteogenesis after AOX1 overexpression. The mRNA expression levels were normalized to that of 18S ribosomal RNA.

To clarify the role of AOX1 in osteogenic differentiation, plasmid transfection was used to efficiently overexpress AOX1 of third-generation hBMSCs. AOX1 expression was quantified by qRT-PCR after infection. Compared with the Ctrl-OE (control-overexpression) group, the mRNA expression of AOX1 was increased in the AOX1-OE (AOX1-overexpression) group (Figure 7E).

### AOX1 Overexpression Increased the Levels of Osteo-Specific Genes

To evaluate the effect of AOX1 overexpression in osteogenic differentiation, the levels of osteo-specific genes including the runt-related transcription factor 2 (RUNX2), osteocalcin (OCN), osteopontin (OPN), and osterix (specificity protein 7, SP7) were determined by quantitative real-time PCR (qRT-PCR). qRT-PCR analysis revealed that RUNX2, OCN, OPN, and SP7 mRNA levels were significantly higher in the AOX1 overexpression group than in the control-overexpression group at day three(*p*<0.05, Figures 7A–D).

## Discussion

Mesenchymal stem cells have been proved to be ideal seed cells for bone tissue engineering and are closely associated with bone defects fracture nonunion and osteoporosis. Better understanding of the molecular mechanism in osteogenesis will enable researchers to design suitable targets for more effectively inducing bone tissue regeneration and treat related diseases. Microarray technology enables us to explore the genetic alterations and identify novel biomarkers in mesenchymal stem cells osteogenesis.

In this study, we performed differential expression analysis between hBMSCs and osteogenically induced samples and identified differentially expressed genes. These genes are found to be significantly associated with osteoblast-related pathways, suggesting a significant value of these genes in regulating osteogenic pathways. The co-expression and PPI network analyses show that AOX1 is a newly discovered potential regulator in osteogenic differentiation of hBMSCs. The GO analysis and GSEA among different AOX1 groups show that osteoblast-related pathways are significantly associated with AOX1 expression.

To verify the gene AOX1 associated with osteogenic differentiation, we performed preliminary verification in hBMSCs. We report here that significant upregulation of AOX1 during the osteogenic differentiation of hBMSCs through the results of PCR and Western blotting. This study revealed that AOX1 overexpression significantly promotes osteogenesis and increases the expression of key genes that regulate the induction of osteogenesis, thereby clearly indicating that AOX1 plays an important role in osteogenic differentiation. As part of a study that was directed toward elucidating the mechanism of action of AOX1, expression of ROS, involved in the osteogenic differentiation (Tao et al., 2020), thereby clearly indicating that AOX1 plays an important role in osteogenic differentiation.

AOX1 is a molybdo-flavoenzyme with complex evolutionary profile, catalyzing the oxidation of a wide variety of substrates (Garattini and Terao, 2012), which is involved in electron transfer (Coelho et al., 2015). The important aspect of AOX1 is that it contributes to the endogenous production of H_2_O_2_ as reactive oxygen species (ROS), thereby regulating intracellular signaling pathways and determining cell proliferation, apoptosis, migration, and differentiation (Kundu et al., 2007; Gough and Cotter, 2011). It is currently believed that absolute levels of ROS is harmful under pathological conditions, whereas moderately elevated ROS have beneficial effects on osteogenic differentiation (Guzik and Harrison, 2006; Mandal et al., 2011; Li et al., 2017; Rossi et al., 2017; Zhu et al., 2018; Gardin et al., 2020; Khalid et al., 2020; Tao et al., 2020; Li et al., 2021). ROS enables induction of FOXO activation, which promotes bone formation in part by modulating the differentiation of osteoblastic cells (Ambrogini et al., 2010) and is also known to suppress the expression and transcriptional activity of PPARγ (a potent repressor of osteoblastogenesis) (Armoni et al., 2006). FOXO1 acts upstream of RUNX2 induction and plays an important role in promoting osteogenic differentiation and suppressing proliferation in differentiating cells (Siqueira et al., 2011).

AOX1 is one of the key enzymes of tryptophan catabolism and loss of AOX1 may lead to the accumulation of kynurenine and NADP (Vantaku et al., 2020). Meanwhile, kynurenine impacts MSCs and causes age-related bone loss (Anaya et al., 2020). AOX1 also contributes to ATRA biosynthesis (Zhong et al., 2021). ATRA-induced osteogenic differentiation of mesenchymal stem cells and its ability to influence osteogenic differentiation has been observed in numerous cell systems (Zhang et al., 2016; Ahmed et al., 2019; Weng et al., 2019).

However, the underlying molecular mechanisms accounting for the role of AOX1 in regulating osteogenic differentiation in hBMSCs need further research. Moreover, these results also suggest that our method has the ability in discriminating genes with osteogenic differentiation properties and can facilitate the process of discovery of new osteogenic differentiation-related genes.

Although more research is necessary to confirm the detailed mechanism during osteogenic differentiation, the present findings indicate that the gene AOX1 plays an important role in the osteogenic differentiation of stem cells and are thus worthy of further exploration. Furthermore, we developed a novel way for the prediction of osteogenic differentiation-relevant genes, and can thus provide potential biomarkers and targets for osteogenic differentiation of stem cells.

## Method

### Data Collection

Gene expression profiles of hBMSCs and osteogenically induced samples are collected from gene expression omnibus (GEO). Four datasets GSE12266, GSE18043, GSE37558, and GSE80614 are downloaded to identify osteogenic differentiation–related genes. GSE12266 contains four hBMSCs and 12 osteogenically induced samples. GSE18043 contains three hBMSCs and nine osteogenically induced samples (Granchi et al., 2010). GSE37558 contains four hBMSCs and 12 osteogenically induced samples (Alves et al., 2014). GSE80614 contains three hBMSCs and 30 osteogenically induced samples. For probes that did not match any gene, we removed them from the expression profiling matrix. For genes that match multiple probes, we averaged the signal intensity of these probes as the expression of the matched gene. To eliminate the platform-based bias and create an integrated dataset, we combined these four datasets using the combat algorithm, which is implemented using the sva R package. In total, 14 controls and 63 osteogenically induced samples are included in our analysis.

MSigDb is an integrated database of annotated gene sets from different sources. Osteoblast-related pathways are retrieved from this database. To identify gene sets that are associated with osteoblast, we search for related gene sets from all of these annotated names using the keyword osteoblast. As a result, 15 gene sets whose name contains osteoblast are identified as osteoblast-related pathways. Among these pathways, one is associated with the osteoblast transcription factor, nine are associated with osteogenic differentiation, one is associated with osteoblast development, three are associated with osteoblast proliferation, and one is associated with osteoblast signaling.

### Single-Sample Gene Set Enrichment Analysis

Single-sample gene set enrichment analysis (ssGSEA) is used to evaluate the activity of a specific pathway in an individual sample. It calculates an enrichment score of a pathway based on the relative expression of these genes involved in the pathway. In this study, we calculated the enrichment score of these 15 osteoblast-related pathways for all samples. The ssGSEA is conducted using gsva R package (Hänzelmann et al., 2013).

### Differential Expression Analysis

Combined expression profiling dataset is used to identify differentially expressed genes between hBMSCs and osteogenically induced samples. limma R package is then applied to identify these genes differentially expressed between the control and osteogenic induction groups based on BH-adjusted *p*-value and fold change. A gene is identified as upregulated if BH-adjusted *p*-value <0.05 and log-transformed fold change >1, and downregulated if BH-adjusted *p-*value <0.05 and log-transformed fold change <−0.8. The reason why we use −0.8 as the threshold for the identification of downregulated genes is that the threshold −1 will lead to a limited number of downregulated genes. As a result, 25 genes are identified as upregulated and 17 genes are identified as downregulated.

### Construction of the Co-Expressed Network

The Pearson correlation coefficient is used to evaluate the co-expression pattern for these differentially expressed genes. Upregulated genes and downregulated genes are analyzed separately to identify hub genes in the co-expressed network. The correlation matrix is first calculated for upregulated genes. Due to the uncertainty of the threshold to construct a co-expressed network, we construct multiple networks based on the varied correlation threshold from 0.6 to 0.9. After analyzing the hub genes of each co-expressed network, we can create a final set of hub genes based on the frequency of hub identified in a single network. We note that the hub genes identified based on this method are reliable and can indeed capture the topological structure of multiple networks compared with a single network. This method is then applied in downregulated genes to identify hub genes that are associated with the osteogenic process.

### Functional Enrichment Analysis

GO enrichment analysis is used to identify pathways that are associated with AOX1. We first divided samples into high AOX1 expression group and low expression group based on the median expression of AOX1. Differentially expressed genes are then identified between these two groups. The GO enrichment analysis is used to identify pathways that are presented by these differentially expressed genes. BH-adjusted *p*-value <0.05 is considered to be statistically significant. The GO enrichment analysis is conducted using the clusterProfiler R package (Yu et al., 2012).

### Gene Set Enrichment Analysis Between Different AOX1 Groups

Gene set enrichment analysis is used to identify pathways that are associated with the AOX1 group. Differential expression analysis between AOX1 groups has been performed earlier. All genes are ranked based on the fold change between AOX1 groups. The ranked gene list and osteoblast-related pathways are then inputted into the gene set enrichment analysis to identify whether osteoblast-related pathways are enriched in the top or the bottom of the ranked gene list. This analysis is conducted using the clusterProfiler R package.

### Cell Culture, Reagents, and Antibodies

hBMSCs were provided by Cyagen Biosciences (Guangzhou, China), which can differentiate into osteoblasts, adipocytes, and chondrocytes under specific inductive conditions. Adherent hBMSCs were incubated in culture flasks in the hBMSC special growth medium (Cyagen Biosciences, Inc., Guangzhou, China) in a cell incubator contained at 37 °C with 5% CO_2_ and were passaged at nearly 80–90% confluence. Cells from passages 2–6 were used in subsequent experiments. Specific antibodies against glyceraldehyde-3-phosphate dehydrogenase (GAPDH) and AOX1 were purchased from Proteintech Group. Inc. (Chicago, United States).

### Osteogenic Differentiation Protocol

hBMSCs were cultured in the special growth medium (Cyagen Biosciences, Guangzhou, China) and 100 IU/ml penicillin/streptomycin) in six- or twelve-well cell culture plates at a density of 3 × 10^4^/cm^2^ and incubated for 48 h at 37 °C under 5% CO_2_. Subsequently, the cells were cultured in osteogenic induction medium (L-DMEM with 10% FBS, 100 IU/ml penicillin/streptomycin, 100 nM dexamethasone, 0.2 mM ascorbic acid, and 10 mM *β*-glycerophosphate). The cells were maintained by the replacement of fresh osteogenic induction medium every 2–3 days.

### Plasmid Transfection

AOX1 plasmid transfection and negative control plasmid were used for overexpression experiments (Genomeditech). hBMSCs were prepared at 50–60% confluence in six-well plates. The transfection mixture was prepared by adding 200 μL Opti-MEM containing 4 μL TransMate reagent (GenePharma) to 200 μL Opti-MEM containing 0.66ug AOX1 plasmid and incubating for 10 min at room temperature. The transfection mixture was added in six-well plates containing 800ul α-MEM with FBS. After transfection for 4 h, the medium was changed to the growth medium.

### RNA Extraction and Quantitative RT-PCR

Levels of the osteoblast formation gene expression were measured using quantitative RT-PCR (qRT-PCR). hBMSCs (3 × 104 cells/cm2) were cultured in six-well plates with the medium. Total cellular RNA was isolated and measured using the RNAiso reagent (Takara Bio, Kusatsu, Japan) and NanoDrop 2000. The absorbance of the samples at 260 nm was calculated in accordance with the manufacturer’s instructions (Thermo Fisher Scientific, MA, United States). Total RNA was reverse-transcribed into complementary DNA (cDNA) in a 20 μL reaction volume (Takara). A total of 2 μL cDNA was used as the template with Power SYBR^®^ Green PCR Master Mix (Takara), and qRT-PCR was performed in triplicate by the ABI 7500 system (Thermo Fisher Scientific); 18S was used as housekeeping genes and each reaction was repeated three times independently. GenePharma synthesized the primer AOX1 and Sangon Biotech (Shanghai, China) synthesized the other primers used in this experiment. Primer sequences are listed in Table 1. The qRT-PCR reaction was at 95°C for 30 s, followed by 45 cycles at 95°C for 5 s, and 60°C for 30 s. The expression levels of all of the genes were evaluated by the 2^−△△Ct^ method.

**TABLE 1:** Sequences of primers for real-time quantitative PCR analysis.

	Primer sequence, 5–3′	
Gene	Forward	Reverse
*AOX1*	CGT​GTT​GGT​GGA​GCG​TTT​G	ATC​GTT​CAT​GAA​TCC​AGC​TTT​GT
*18S*	CGC​CGC​TAG​AGG​TGA​AAT​TC	TTG​GCA​AAT​GCT​TTC​GCT​C
OCN	CAC​TCC​TCG​CCC​TAT​TGG​C	CCCTCCTGCTTGGACA CAAAG
*RUNX2*	ACT​TCC​TGT​GCT​CGG​TGC​T	GAC​GGT​TAT​GGT​CAA​GGT​GAA
*OPN*	CTCCATTGACTCGAACGACTC	CAGGTCTGCGAAACTTCT TAGAT

*SP7*	AGC​CCA​TTA​GTG​CTT​GTA​AAG​G	CCT​CTG​CGG​GAC​TCA​ACA​AC

### Western Blotting Analysis

Cells were lysed for 30 min on ice in a RIPA buffer containing phosphatase and protease inhibitor cocktails (Beyotime, Shanghai, China). The centrifugation to clear the lysates and collect the supernatants was set at 14,000 rpm for 10 min at 4°C. Equal amounts of proteins were separated by 10% sodium dodecyl sulfate polyacrylamide gel electrophoresis and then transferred to a polyvinylidene fluoride membrane (Millipore, Shanghai, China) which was then probed with the primary antibodies. The membranes were then blocked for 1 h at room temperature in Tris-buffered saline containing 10% non-fat milk and 0.1% Tween. Subsequently, the membranes were incubated with primary antibodies overnight at 4°C. After washing with 0.1% Tween in Tris-buffered saline for three times and incubation with horseradish peroxidase–conjugated secondary antibodies (anti-rabbit; Beyotime) for 1 h at room temperature, proteins were visualized by an enhanced chemiluminescent detection reagent (MilliporeSigma) and an XRS chemiluminescence detection system (Bio-Rad Laboratories, Hercules, CA, United States).

### Statistical Analysis

Statistical analysis was performed using GraphPad Prism v.7.0 (GraphPad Software, San Diego, CA, United States). All experiments were performed at least in triplicate. Data are presented as the mean ± SD. Statistical significance was determined using a two-tailed Student’s *t* test when comparing two groups, and one-way ANOVA followed by Tukey’s *post hoc* test was used when comparing more than two groups. A value of *p-* ≤ 0.05 was considered to indicate statistical significance.

## Data Availability

The datasets presented in this study can be found in online repositories. The names of the repository/repositories and accession number(s) can be found in the article/Supplementary Material.
